# Supplementary material for: Stearic acid blunts growth-factor signaling via oleoylation of GNAI proteins
Source: Nat Commun. 2021 Jul 28;12:4590. doi: 10.1038/s41467-021-24844-9 (PMC8319428; doi:10.1038/s41467-021-24844-9)
Supplement: Supplementary file 1 — Supplementary Information [file 41467_2021_24844_MOESM1_ESM.pdf]

Supplementary Information

Stearic acid blunts growth-factor signaling via oleoylation of GNAI proteins

Nůsková et al.

Table of Contents

1.Cover Page... p. 1

2. Supplementary Figures with Legends.. p. 2

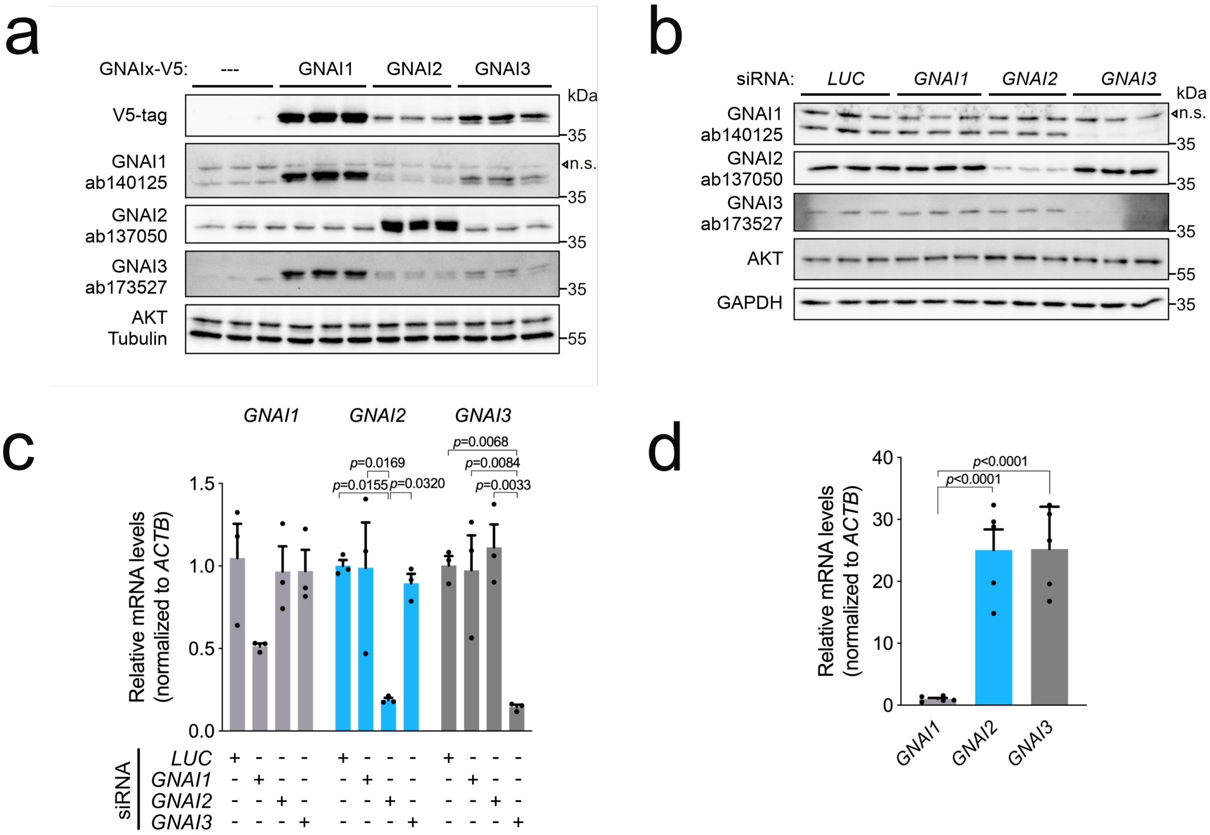

Supplementary Figure 1: Characterization of GNAI antibody specificity in MCF7 cells.

(a-b) The specificity of GNAI antibodies was tested using cells overexpressing V5-tagged GNAI isoforms in **a** and knockdown cells in **b**. Note that the overexpressed GNAI proteins run slightly higher than the endogenous proteins because of their V5 tags. Due to their high homology, GNAI1- and GNAI3-specific antibodies recognize both GNAI1 and GNAI3, as well as GNAI2 weakly. In knockdown cells, however, the signal from GNAI1 and GNAI3 antibodies decreases only upon *GNAI3* knockdown, indicating they are both detecting endogenous GNAI3. This is likely because the expression of *GNAI3* is higher (>20-fold) than the expression of *GNAI1* as shown at the mRNA level in **d**. In contrast, the GNAI2 antibody specifically detects GNAI2. Non-specific bands are indicated (n.s.).

(c) To investigate the efficiency of knockdown of different GNAI isoforms, q-RT-PCR was used to determine the expression of *GNAI* mRNA in knockdown cells relative to control cells transfected with luciferase-targeting siRNA. Results are normalized to *ACTB* ($2^{-\Delta\Delta Ct}$ values, n=3 biological replicates).

(d) Gene expression of *GNAI1/2/3* normalized to *ACTB*, relative to *GNAI1* ($2^{-\Delta\Delta Ct}$ values, n=5 biological replicates).

Data are presented as mean±SEM in **c** and **d** and the *p*-values were determined by one-way ANOVA followed by Tukey's post hoc test.

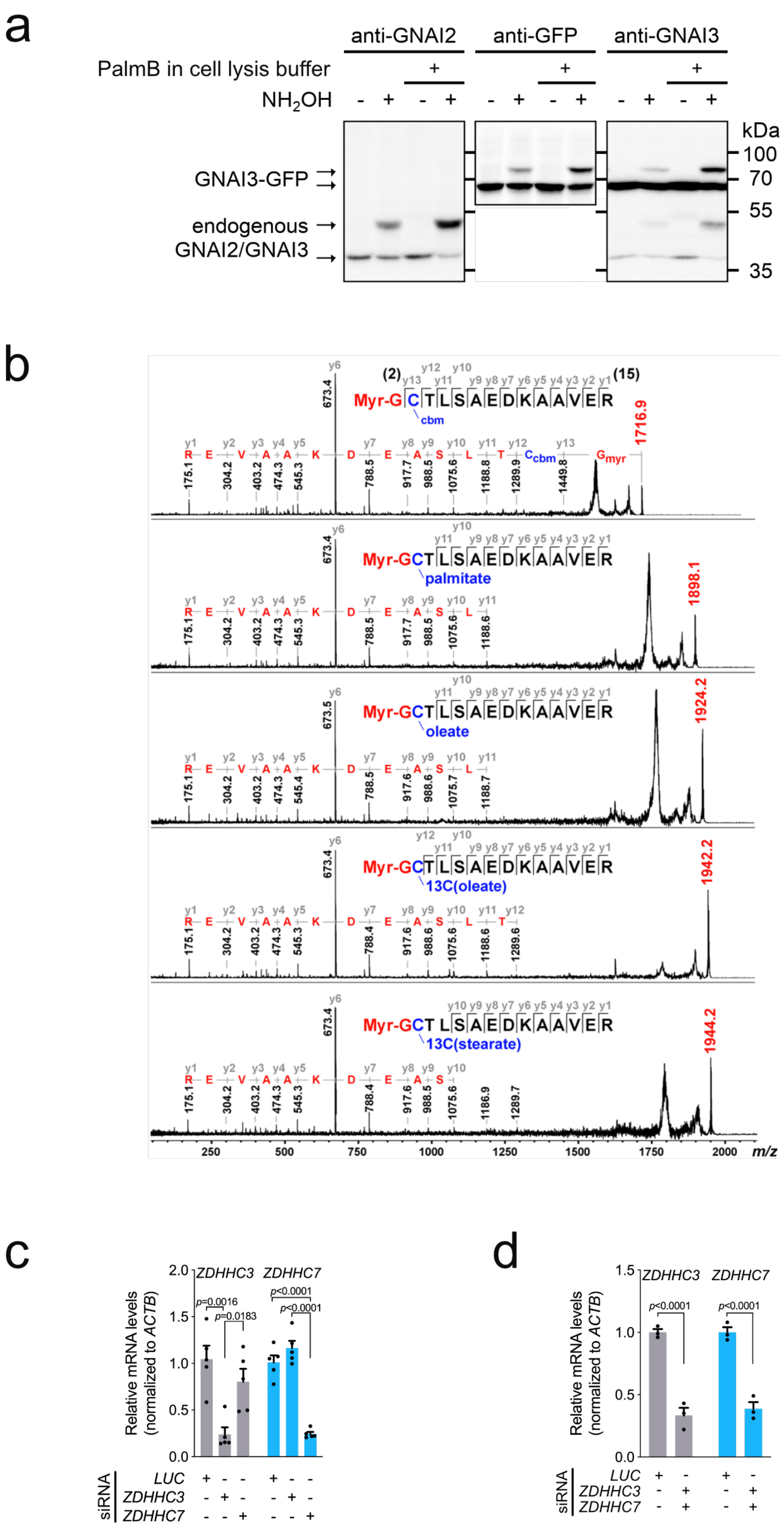

Supplementary Figure 2: When added to the lysis buffer at the time of cell lysis, palmostatin B, an APT1 inhibitor, helps preserve acylation of endogenous GNAI proteins and of overexpressed GNAI3-GFP.

(legend continues on next page)

(a) Cells stably overexpressing GNAI3-GFP were lysed in the lysis buffer used for GFP pulldown (20 mM Tris-Cl, 800 mM NaCl, 0.5 mM EDTA, 1 % NP-40, pH 7.5, supplemented with a protease inhibitor cocktail) and kept on ice for 2 h, which is the time applied for GFP pulldown. Afterwards the acyl-PEG exchange assay was performed and the acylation status of both endogenous GNAI proteins and GNAI3-GFP was investigated by observing the mass shift induced by the acyl-PEG exchange. The acylation was mostly lost unless palmostatin B (PalmB) was added to the lysis buffer (final concentration 250 µM). Representative of two independent experiments.

(b) Tandem MS analysis of the detected N-terminal peptides described in main **Figure 3c-f**. Parent ions with *m/z* values of 1716.9, 1898.1, 1924.2, and 1942.2 (in red) were subjected to fragmentation analysis. Detected series of daughter C-terminal y-type ions confirmed the amino acid identity and fatty acid patterns of the respective peptides depicted above each MS/MS spectrum. Fragmentation of the 1944.2 *m/z* species is not technically feasible due to the larger nearby 1942.2 *m/z* peak. The N-terminal endogenously myristoylated glycine is indicated (Myr-G).

(c-d) Efficiency of *ZDHHC3* and *ZDHHC7* knockdowns characterized by q-RT-PCR in single knockdowns in **c** and *ZDHHC3*+*ZDHHC7* double knockdown in **d**. The mRNA levels of *ZDHHC* genes in knockdown cells are normalized to *ACTB* and results are shown relative to control cells transfected with luciferase-targeting siRNA ($2^{-\Delta\Delta Ct}$ values, n=5 biological replicates in **c** and n=3 biological replicates in **d**). In **c**, data are presented as mean±SEM and the *p*-value by one-way ANOVA followed by Tukey's post hoc test is indicated. In **d**, data are presented as mean±SEM and the *p*-value determined by two-sided t-test with correction for multiple comparisons using the Holm-Sidak method is indicated.

(Figures continue on next page)

a

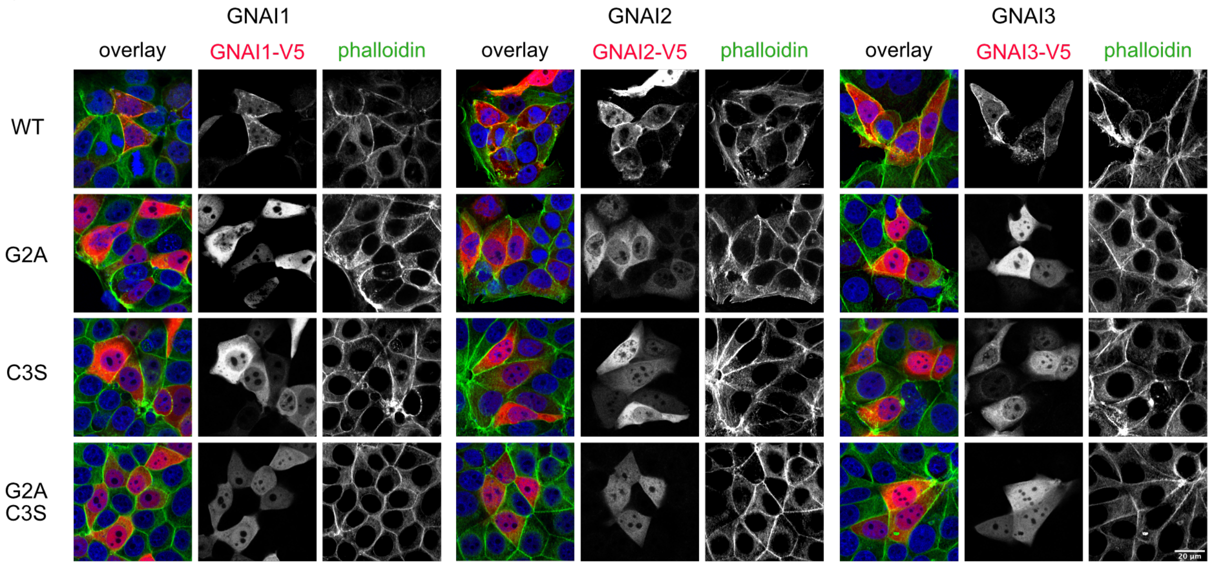

b

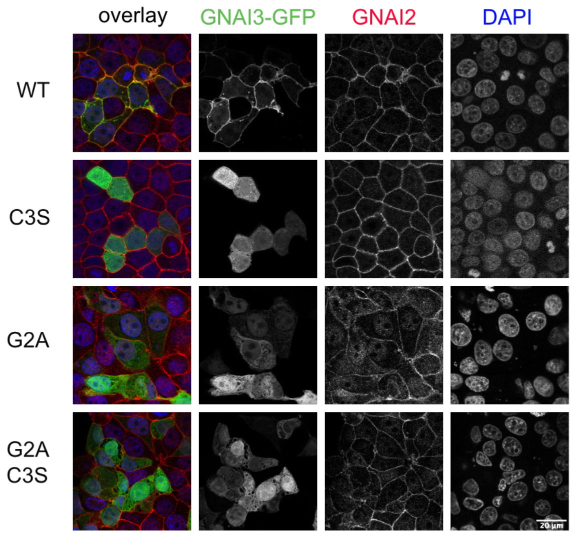

Supplementary Figure 3: S-acylation of GNAI proteins is required for plasma membrane association.

(a-b) S-acylation of V5-tagged (**a**) or GFP-tagged (**b**) GNAI proteins is required for their membrane localization. Unlike wild-type (WT) GNAI proteins, GNAI mutants lacking S-acylation (Gly2Ala, Cys3Ser, Gly2Ala/Cys3Ser) do not localize to the plasma membrane, detected by immunostaining. Representative of two biological replicates. (**a**) Red: anti-V5, green: phalloidin-Alexa Fluor 488, blue: DAPI, scale bar 20 µm. (**b**) Green: GFP, red: endogenous GNAI2, blue: DAPI. Scale bar – 20 µm.

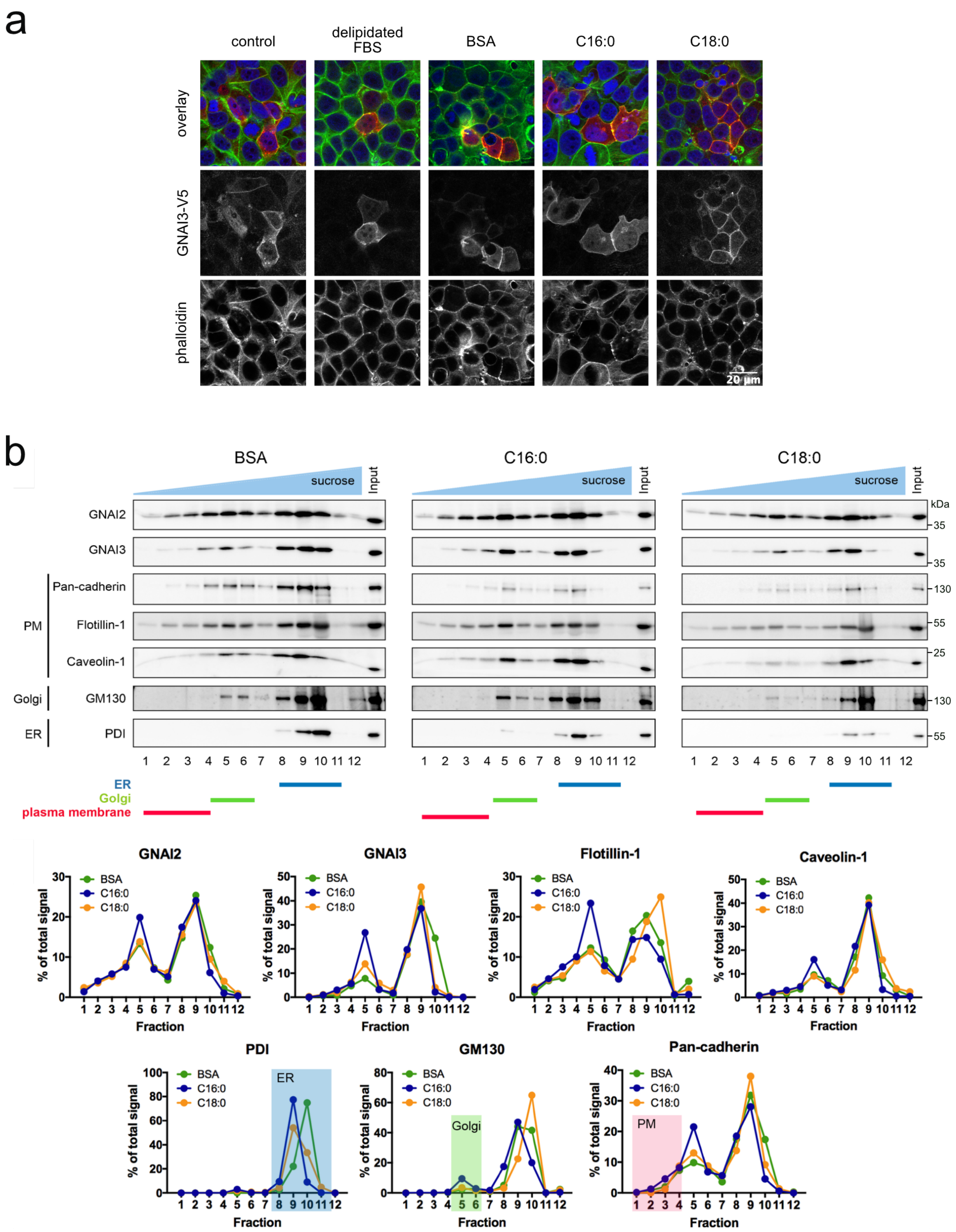

Supplementary Figure 4: Exposure of cells to C18:0 does not interfere with localization of GNAI proteins to the plasma membrane.

(a) GNAI proteins localize to the plasma membrane both in the presence and absence of exogenous C16:0 and C18:0 in the medium. Cells were incubated with standard medium (control), medium containing delipidated FBS, or control

medium supplemented with C16:0 or C18:0 (final concentration of BSA-conjugated fatty acids 100 µM, 24 h treatment). Representative of two biological replicates. Red: GNAI3-V5 detected with anti-V5 antibody, green: phalloidin-Alexa Fluor 488, blue: DAPI, scale bar 20 µm.

(b) Separation of cell membrane compartments using sucrose gradients shows that the presence or absence of C18:0 does not significantly affect the relative levels of GNAI proteins in different membrane compartments (ER, endoplasmic reticulum; Golgi, Golgi apparatus; PM, plasma membrane). Top panel: immunoblots. Bottom panels: quantifications. Cells were incubated with 100 µM BSA-conjugated fatty acids for 24 h prior to lysis.

(Figures continue on next page)

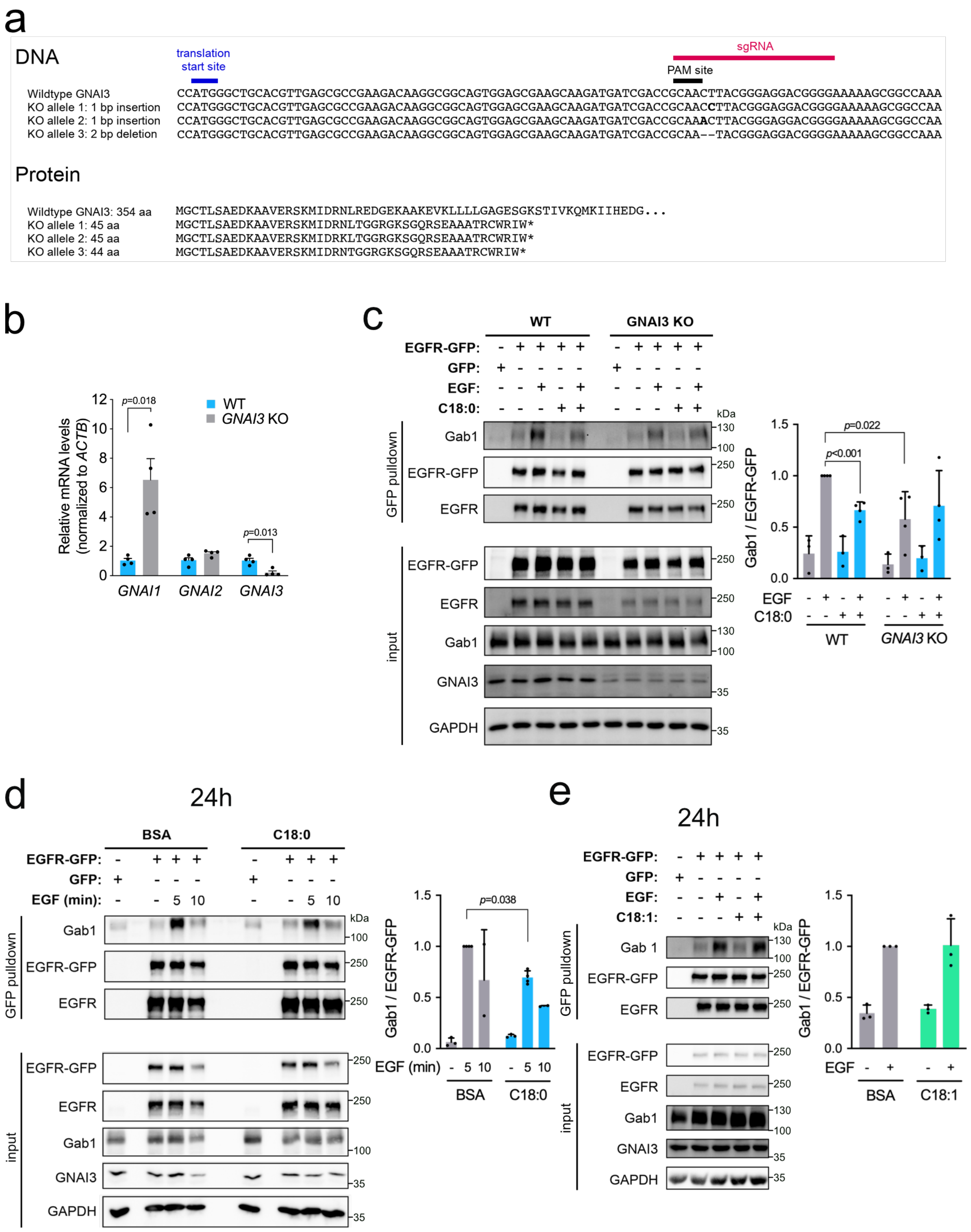

Supplementary Figure 5: Exposure of cells to C18:0 blunts Gab1 recruitment in MCF7 cells.

(a) Molecular characterization of *GNAI3* knockout MCF7 cells. MCF7 cells have 3 copies of the *GNAI3* locus, all of which are edited to yield out-of-frame coding sequences and premature stop codons.

(legend continues on next page)

(b) *GNAI3* knockout cells have compensatory, elevated expression of *GNAI1*. Expression levels of *GNAI1*, *2* or *3* were quantified by q-RT-PCR, normalized to *ACTB*. Presented values represent mean±SEM of four biological replicates and the *p*-values determined by two-sided t-test with correction for multiple comparisons using the Holm-Sidak method are indicated.

(c) Knockout of *GNAI3* blunts the effect of C18:0 (100 μM, 3 h) on Gab1 recruitment to EGFR-GFP upon EGF stimulation (50 ng/mL for 5 min). Quantification: mean±SEM of four biological replicates, the *p*-values determined by two-sided t-test with correction for multiple comparisons using the Holm-Sidak method are indicated.

(d-e) The EGF (50 ng/mL, 5 min)-induced interaction of EGFR-GFP with Gab1 is still blunted 24 hours after adding C18:0 (**d**) but not C18:1 (**e**) (both 100 μM) to the medium of MCF7 cells. In **d**, n=4 for 5 min and n=2 for 10 min treatment. In **e**, n=3 biological replicates. Presented values represent mean±SEM and the *p*-values determined by two-sided t-test with correction for multiple comparisons using the Holm-Sidak method are indicated.

(Figures continue on next page)

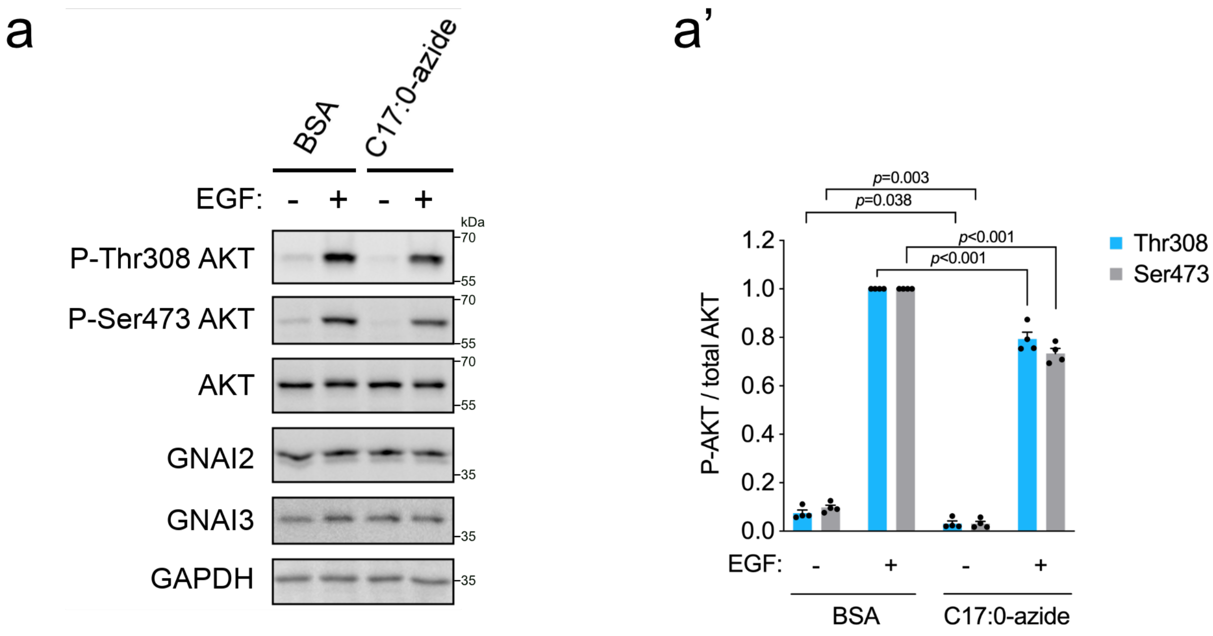

Supplementary Figure 6: C17:0-azide is functionally equivalent to C18:0

(a-a’) C17:0-azide blunts AKT phosphorylation in response to EGF. Cells were treated with BSA-conjugated C17:0-azide (100 µM, 24h) or with BSA as a vehicle control. AKT phosphorylation was investigated upon EGF stimulation (50 ng/mL; 10 min treatment). Quantified in a’: Data represent mean±SEM of four biological replicates and the *p*-values determined by two-sided t-test with correction for multiple comparisons using the Holm-Sidak method are indicated.

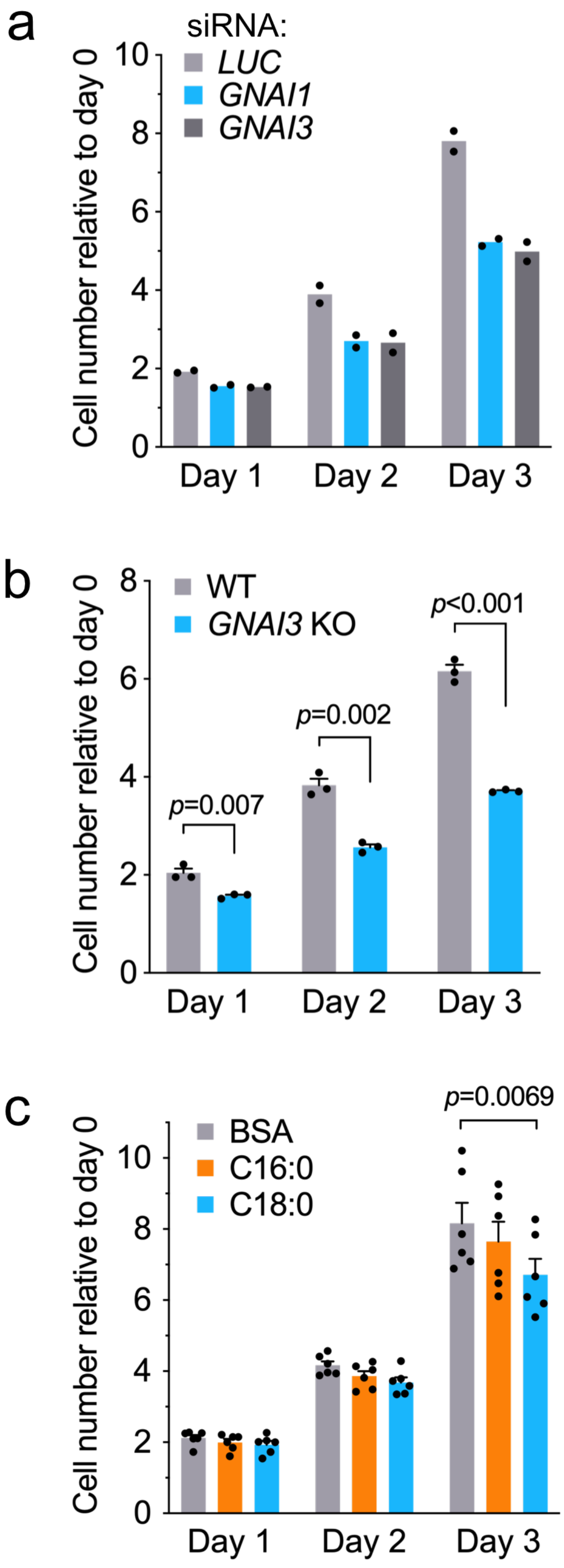

Supplementary Figure 7: Exposure of MCF7 cells to C18:0 reduces their proliferation rate.

(legend continues on next page)

(a) Knockdown of *GNAI1* or *GNAI3* reduces MCF7 proliferation.

(b) *GNAI3* knockout MCF7 cells have reduced proliferation compared to control cells. As a control, the parental cell line (WT) from which the *GNAI3* KO cell line was derived, is used. Data represent mean±SEM of three biological replicates Presented values represent mean±SEM of four biological replicates and the *p*-values determined by two-sided t-test with correction for multiple comparisons using the Holm-Sidak method are indicated.

(c) Exposure of MCF7 cells to C18:0 but not C16:0 reduces their proliferation rate, assayed by Hoechst 33258. Cells were treated with BSA-conjugated 100 μM C18:0 or C16:0 or only BSA as a vehicle control. Data represent mean±SEM of six biological replicates and the *p*-value by two-way ANOVA followed by Tukey's post hoc test is indicated.

(Figures continue on next page)

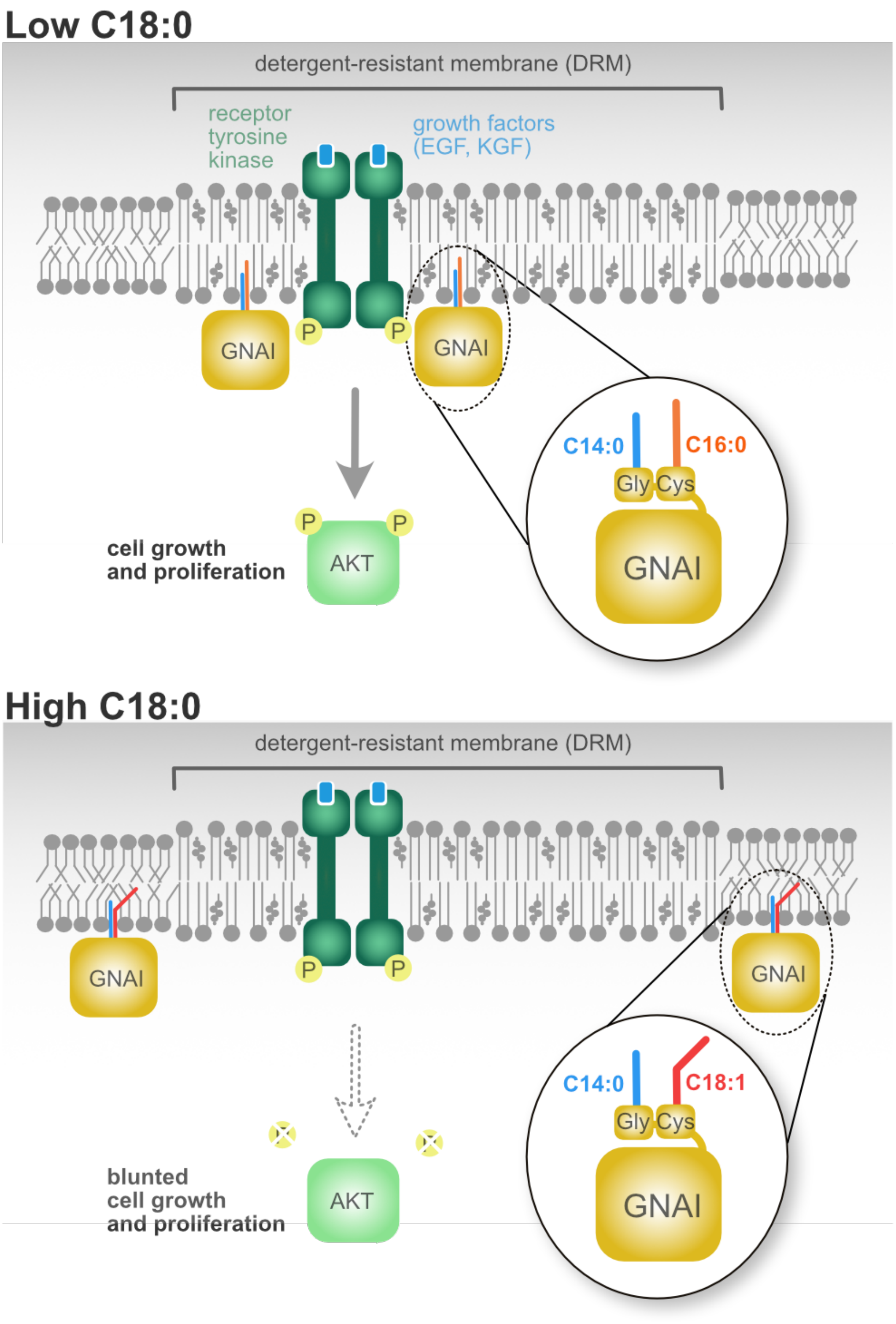

Supplementary Figure 8: Schematic diagram illustrating the findings of this manuscript.
When cells are exposed to C18:0 or C18:1, this increases the relative proportion of GNAI proteins that are oleoylated on Cys3, rather than palmitoylated. This shifts GNAI proteins out of detergent resistant membrane fractions of the cell membrane, leading to reduced activation of EGFR signaling, including AKT phosphorylation.
