# Supplementary material for: Stearic acid blunts growth-factor signaling via oleoylation of GNAI proteins
Source: Nat Commun. 2021 Jul 28;12:4590. doi: 10.1038/s41467-021-24844-9 (PMC8319428; doi:10.1038/s41467-021-24844-9)
Supplement: Supplementary file 3 — Description of Additional Supplementary Files [file 41467_2021_24844_MOESM3_ESM.pdf]

Description of Additional Supplementary Files

File Name: Supplementary Data 1
Description: Catalog numbers and sequences of siRNAs used in this study.

File Name: Supplementary Data 2
Description: Sequences of primers used in this study.

File Name: Supplementary Data 3
Description: additional details regarding acyl-CoA measurements by LC-MS/MS, including instrument settings, raw data and calibration curves.
